# Computational Analysis of the ESX-1 Region of *Mycobacterium tuberculosis*: Insights into the Mechanism of Type VII Secretion System

**DOI:** 10.1371/journal.pone.0027980

**Published:** 2011-11-30

**Authors:** Chandrani Das, Tarini Shankar Ghosh, Sharmila S. Mande

**Affiliations:** Bio-sciences R& D Division, Tata Consultancy ServicesInnovation Labs, Tata Consultancy Services Ltd, Hyderabad, Andhra Pradesh, India; University of Hyderabad, India

## Abstract

Type VII secretion system (T7SS) is a recent discovery in bacterial secretion systems. First identified in *Mycobacterium tuberculosis*, this secretion system has later been reported in organisms belonging to the Actinomycetales order and even to distant phyla like Firmicutes. The genome of *M. tuberculosis* H37Rv contains five gene clusters that have evolved through gene duplication events and include components of the T7SS secretion machinery. These clusters are called ESAT-6 secretion system (ESX) 1 through 5. Out of these, ESX-1 has been the most widely studied region because of its pathological importance. In spite of this, the overall mechanism of protein translocation through ESX-1 secretion machinery is not clearly understood. Specifically, the structural components contributing to the translocation through the mycomembrane have not been characterized yet. In this study, we have carried out a comprehensive in silico analysis of the genes known to be involved in ESX-1 secretion pathway and identified putative proteins having high probability to be associated with this particular pathway. Our study includes analysis of phylogenetic profiles, identification of domains, transmembrane helices, 3D folds, signal peptides and prediction of protein-protein associations. Based on our analysis, we could assign probable novel functions to a few of the ESX-1 components. Additionally, we have identified a few proteins with probable role in the initial activation and formation of mycomembrane translocon of ESX-1 secretion machinery. We also propose a probable working model of T7SS involving ESX-1 secretion pathway.

## Introduction

Bacterial secretion systems are responsible for the export of virulence factors either to the extracellular environment or directly into the host cell and thus, play a crucial role in the virulence of a pathogen [1]. Currently, seven categories of secretion systems (Type I to Type VII) have been identified in bacteria [1]–[3]. These secretion systems not only differ in terms of the secreted effector molecules, but also in their structural components. While Type I, II, III, V and VI have been found to be typically associated with Gram-negative bacteria, Type IV is found in both Gram-positive as well as Gram-negative bacteria. The most recently categorized Type VII secretion system (T7SS) is observed to be present in the Gram-positive species, mostly belonging to the Actinomycetales order [4]. A few components related to the T7SS have also been identified in some species belonging to the phylum Firmicutes [4]–[6]. The T7SS was first identified in the pathogenic organism *Mycobacterium tuberculosis* H37Rv and the corresponding gene clusters were later referred to as the ESX (ESAT-6 Secretion System) regions [2]–[4], [7]. The T7SS has been shown to secrete proteins lacking classical signal peptides in contrast to that observed in Type II, IV and V secretion systems. Furthermore, most of the proteins secreted by T7SS follow a pairwise dependency, both for secretion and function [8].

The first ESX region (ESX-1) was discovered during the comparative genomic analysis of the attenuated strain *Mycobacterium bovis* Bacille Calmette-Guerin (BCG) and other pathogenic mycobacterial species [9]. It was observed that the genome of the *M. bovis* BCG had ten different regions of deletion (RD1-RD10) as compared to that of *M. tuberculosis*. Out of these, the region RD1, containing a total of nine genes including the genes encoding the secreted antigens CFP-10 and ESAT-6, was observed to be especially responsible for virulence [10], [11]. Restoration of this region not only enabled the secretion of ESAT-6, but also led to increased virulence in *M. bovis* BCG [10], implicating the role of the genes in RD1 region in the virulence of the bacteria. Concurrently, several computational studies have attempted to predict the functional role of the genes encoded in the RD1 region [12], [13]. It was predicted that, this region contained genes encoding ATP dependent motors, a number of transmembrane proteins, a protease and secretory proteins [13]. Furthermore, most of the genes encoded in this region lacked significant similarity to previously characterized proteins. Based on these observations, Pitius et al. (2001) hypothesized that, the RD1 region (ESX-1) in the *Mycobacterium* genus encodes components of a novel secretion system. Apart from ESX-1, four other gene clusters (ESX-2 to ESX-5) were subsequently discovered in *M. tuberculosis* H37Rv. The ESX gene clusters have been shown to have evolved through gene duplication events in the order: ESX-4, ESX-1, ESX-3, ESX-2 and ESX-5 [13]. Several studies have provided evidences for the involvement of one or more of these regions (especially ESX-1 and ESX-5) in pathogenesis and macrophage escape [2]–[4], [7], [14], [15]. A majority of these studies have focused specifically on the ESX-1 region. One of the first characterized substrates of the ESX-1 secretion system was the CFP-10 (Rv3874)/ESAT-6 (Rv3875) pair [2]. The 3D structure of the complex of these two substrates showed that, both CFP-10 and ESAT-6 adopt helix-turn-helix folds and interact with each other forming a tight four-helix-bundle [16]. Five such CFP-10/ESAT-6 gene pairs were identified in the 5 ESX regions [13]. Apart from these five pairs (one pair in each ESX region), six additional CFP-10/ESAT-6 like gene pairs were also identified outside the ESX regions [13]. Interestingly, protein pairs with similar properties were observed not only in other Actinobacterial species, but also in distant phyla like Firmicutes [5]. The proteins constituting these pairs had certain conserved structural properties like length of approximately 100 residues forming probable helix-turn-helix structures and containing a conserved WXG motif in the region linking the two helices. Such pairs were subsequently referred to as belonging to the WXG100 superfamily [5].

Previous studies have analyzed one or more components of ESX-1 region (covering genes Rv3866-Rv3883c) and predicted probable models for the translocation of proteins [4], [15], [17], [18]. The overall model of the mechanism emerging from these studies is as follows. The C-terminal end of CFP-10 contains a probable signal sequence, that interacts with the FtsK domain containing protein Rv3871 [3], [7], [19], [20]. Subsequently, Rv3871 interacts with Rv3870 and recruits the CFP-10/ESAT-6 pair to the probable inner membrane pore (Rv3877), which then translocates this pair through the inner membrane. Two other proteins, namely, EspA (Rv3616c) and EspC (Rv3615c), have been associated with this secretion pathway. These two proteins have been shown to be secreted along with CFP-10/ESAT-6 pair in a mutually dependent manner [8], [21]. In addition, a few genes located inside ESX-1 have also been shown to be essential for the expression or secretion of these proteins. These include Rv3866, Rv3868, Rv3869, PE35 (Rv3872) and MycP1 (Rv3883c) [2], [7], [22]. However, the probable mechanism by which these components aid or modulate the secretion is still ill defined. The focus of most of the above studies has been on the inner membrane translocation machinery. However, in addition to the inner membrane, mycobacterial species have a complex mycolic acid rich outer membrane, referred to as the mycomembrane. The components of the ESX-1 secretion machinery, constituting the translocon through the mycomembrane are still unknown.

In this study, we have performed a detailed in silico characterization of the proteins encoded by the genes located in ESX-1 cluster (from 14 different mycobacterial species) using a combination of bioinformatics analysis and known information from previously reported studies (experimental and computational). Our analysis suggests functional roles for various components associated with the ESX-1 secretion pathway. Based on these results, we also propose a probable working mechanism of Type VII secretion system in *M. tuberculosis*.

## Results

### Orthology search and analysis of phylogenetic profiles

Proteins interacting with each other or belonging to same biochemical pathway are known to be conserved across all bacterial species possessing that particular pathway. In order to identify the interaction patterns between ESX-1 components and also to identify additional proteins that may act as regulators or as accessory components of this secretion machinery, we adopted the phylogenetic profile based approach. For this purpose, orthologs of each protein belonging to *M. tuberculosis* H37Rv were first identified across other mycobacterial species. The orthology search of ESX-1 components of *M. tuberculosis* H37Rv against 13 mycobacterial species identified three species, which did not have orthologs corresponding to any of the 17 ESX-1 components. These species corresponded to *Mycobacterium avium*, *Mycobacterium ulcerans* and *Mycobacterium abscessus*. The remaining ten species were observed to contain orthologs corresponding to at least nine out of the 17 ESX-1 components. Table S1 gives the number of orthlogs corresponding to the ESX-1 components identified in the various mycobacterial species. While *M. bovis* had orthologs corresponding to all the 17 components, other species lacked orthologs corresponding to Rv3876 and Rv3879c. In addition, orthologs of Rv3881c (EspB) were observed to be present only in *M. bovis* and *Mycobacterium marinum*. These results suggest that the three proteins (Rv3876, Rv3879c and EspB) may not be an integral or essential components of the secretion machinery and probably perform organism specific functions.

**Figure 1:**
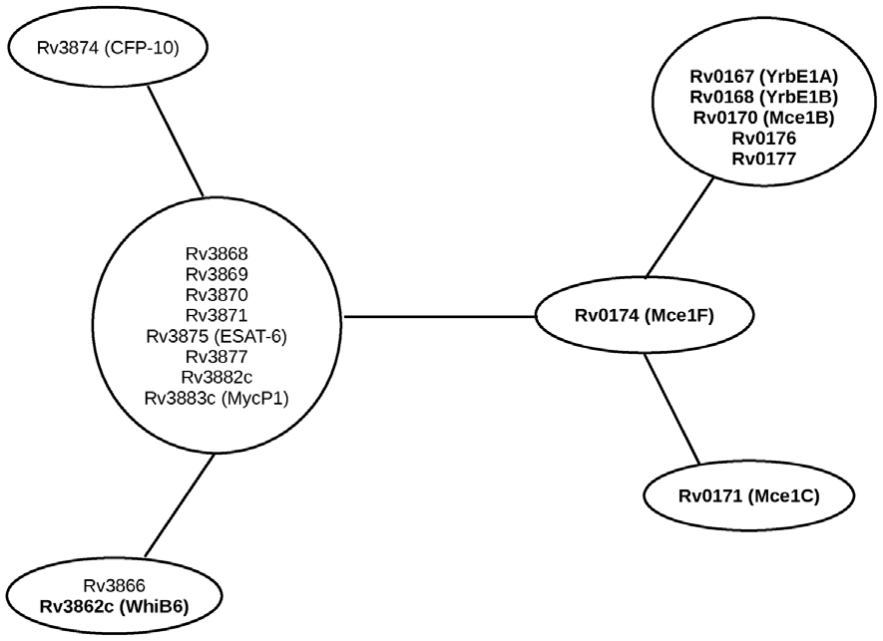
Relationships between the phylogenetic profiles of the probable proteins involved in ESX-1 secretion pathway. Each node (represented as an oval) denotes proteins having identical phylogenetic profiles. Edge between two nodes is equivalent to one bit difference between the phylogenetic profiles of the corresponding nodes. The bit difference between any two nodes is equal to the number of edges required to travel between the two nodes. Proteins which lie outside the ESX-1 region, but were identified to have similar phylogenetic profiles to one or more of the ESX-1 components are highlighted in bold.

Phylogenetic profiles represent the presence/absence of orthologs corresponding to a set of proteins in organisms under study. Proteins involved in the same pathway show co-evolution and are thus likely to possess similar phylogenetic profiles [23]. Hence, we have used the phylogenetic profile based analysis in order to identify putative structural or regulatory proteins involved in ESX-1 secretion pathway. Comparison of the phylogenetic profiles of ESX-1 components indicated eight components having identical profiles. Apart from this major cluster, two of the ESX-1 components were observed to be located in the neighborhood of this cluster, suggesting their functional relatedness. The neighborhood of a protein (or a protein cluster) can be defined in terms of bit difference. The bit difference between any two proteins (or protein clusters) is defined as the number of positions where their phylogenetic profiles differ. In other words, this denotes the number of species wherein one of the proteins (or protein clusters) has an ortholog whereas the other does not. The bit difference thus provides a quantitative measure of the degree of similarity in the co-evolution trends of the compared proteins (or protein clusters). In the current study, the set of proteins constituting the neighborhood of the major cluster were identified as those whose phylogenetic profiles had a bit difference of less than or equal to three with that corresponding to the major cluster. Details of the proteins present in the major cluster as well as those located in its neighborhood are discussed below and are summarized in Figure 1.

Out of the 17 ESX-1 components, the major cluster contained eight components (present in 11 organisms including *M. tuberculosis* H37Rv), namely, Rv3868, Rv3869, Rv3870, Rv3871, Rv3875 (ESAT-6), Rv3877, Rv3882c and Rv3883c (MycP1). While the phylogenetic profiles of Rv3866 and Rv3874 (CFP-10) differed from the major cluster by a bit difference of one, their phylogenetic profiles differed from each other by a bit difference of two (Figure 1). Orthologs of Rv3866 and CFP-10 were absent in *Mycobacterium lepreae* and *Mycobacterium vanbaalenii*, respectively. The remaining seven components of ESX-1, namely, Rv3872 (PE35), Rv3873 (PPE68), Rv3876, Rv3879c, EspB, Rv3615c (EspC) and Rv3616c (EspA), did not belong to the neighborhood space of the major cluster. Furthermore, none of them had phylogenetic profiles identical to each other. These results indicate that the above seven ESX-1 components probably have organism-specific functions.

The present study identified additional proteins, not belonging to the ESX-1 region, but having phylogenetic profiles either identical or similar to the profile of some of the ESX-1 components. For example, Rv3862c (WhiB6) had phylogenetic profile identical to that of Rv3866. Apart from this, five proteins, namely, Rv0167 (YrbE1A), Rv0168 (YrbE1B), Rv0170 (Mce1B), Rv0171 (Mce1C) and Rv0174 (Mce1F), corresponding to Mammalian Cell Entry cluster 1 (MCE 1), had phylogenetic profiles similar to that of some of the ESX-1 components. Similarly, two MCE 1-associated proteins (Rv0176 and Rv0177) also showed similarity in their phylogenetic profiles with those of ESX-1 components. The above observations suggest that these MCE and MCE-associated proteins probably have functional relationships with the ESX-1 components and may serve as accessory components of the ESX-1 secretion pathway.

### Domain search

Identification of known functional domains in hypothetical proteins may help in providing valuable information about their functional roles. The search of the ESX-1 components against protein domain databases revealed new information about two hypothetical proteins, namely, Rv3866 and Rv3876 (Table S2). Rv3866 was found to possess a DNA binding domain, suggesting its probable function in transcriptional regulation. Rv3876 was identified to have an FlhG domain. FlhG domain containing proteins have been earlier reported to be involved in negative regulation of flagellar biogenesis in *V. cholerae* [24]. These results indicate a probable role of Rv3866 and Rv3876 in the regulation of the secretion machinery.

### Prediction of transmembrane helices

Identification of ESX-1 components with probable transmembrane (TM) regions is likely to aid in the detection of proteins forming the inner membrane or the mycomembrane transolocon. Identification of TM regions may also help to demarcate the domains of some of the ESX-1 components that are located in the cytoplasmic, periplasmic and/or mycomembrane regions. This may help to gain further insights into the probable mechanism of these components. The cytoplasmic and periplasmic domains of some of the ESX-1 components were identified based on the position and orientation of the TM helices predicted for them. These details are listed in Table S2. The orientation of the TM regions detected in some of the ESX-1 components, namely, Rv3869, Rv3870, Rv3877, Rv3883c (MycP1) and Rv3882c, are in accordance with what has been reported earlier [13].

Rv3869 (480 amino acids) was predicted to contain a single TM helix approximately from residues 40–60, and thus, had a long portion (∼420 aa) in periplasm-like space. Rv3870 was identified with two N-terminal TM helices with a short periplasmic loop. The inner membrane pore forming protein, Rv3877, was predicted to contain either ten or eleven TM helices. Analysis of MycP1 showed a single C-terminal TM helix preceded by a long N-terminal region (∼420 aa) in the periplasm-like space. Rv3882c was predicted to possess two N-terminal TM helices with a short cytoplasmic loop.

### Prediction of 3D folds

The three dimensional structure or fold of a protein is known to provide insights into its probable function. Proteins with similar function often tend to adopt similar folds even when they have very low sequence similarity. The results obtained from the 3D fold analysis indicate probable novel functional roles for seven ESX-1 components, namely, Rv3868, Rv3876, Rv3877, Rv3879c and the secreted proteins Rv3881c (EspB), Rv3616c (EspA) and Rv3615c (EspC). The regions containing these predicted 3D folds (for each of the above components) are illustrated in Figure S1.

Rv3868 was identified to possess an N-terminal TPR (tetratricopeptide repeat) domain. Such TPR domains are known to aid in the assembly of protein complexes [25]. Rv3876 was found to have a fold similar to ParA family proteins, which are known to function as suppressors of *par* operon involved in chromosome partitioning [26]. Analysis with Rv3877 predicted the N-terminal region of the protein to possess a ubiquitin-like domain. Ubiquitin-like domains are known to direct proteins either for degradation or to locations where they control other proteins and cell mechanisms.

The 3D fold analysis of EspB predicted a helix-turn-helix fold at the N-terminal of the protein, similar to that adopted by the PE protein of a previously studied PE/PPE protein complex [27]. Interestingly, the C terminal end of EspB was predicted to have a fold similar to that adopted by the PPE counterpart of this complex, with a WEG tripeptide motif at the position 176–178. This tripeptide motif is similar to the WXG motif observed for the ESAT-6 like family of proteins. The N-terminal region of Rv3879c exhibited a fold similar to fibrinogen, with helical regions followed by coiled-coil regions. In addition, a WXG motif was present in the coil connecting the first two helices. These observations suggest that the N-terminal regions of EspB and Rv3879c together probably form a four-helix-bundle similar to CFP-10/ESAT-6 pair.

Interestingly, the 3D fold analysis on EspC predicted this protein to adopt a fold similar to that of ESAT-6 like proteins (i.e. helix-turn-helix structure). The N-terminal region of EspA exhibited helical regions with WXG motif present in the linker region of the first two helices. This is similar to that observed for CFP-10, ESAT-6, EspB and Rv3879c. The positions of the conserved WXG motifs in EspB, Rv3879c and EspA are shown in Figure S2. The C-terminal region of EspA, on the other hand, showed a triple-helix conformation similar to that adopted by collagen molecules. The 3D folds predicted for each of these ESX-1 components are further summarized in Table S2.

### Signal peptide search

Bacterial secretion systems II, IV and V have been shown to translocate proteins with N-terminal signal sequences, which are recognized by the Sec or Tat translocons [28]–[30]. However, the known substrates of ESX-1 secretion machinery (ESAT-6, CFP-10, EspA, EspC, EspB) have been reported to lack such signal sequences [4]. Besides the secreted proteins, many of the known bacterial outer membrane proteins also possess N-terminal signal sequence. Such proteins are known to contribute to the formation of the outer membrane pore apparatus of other secretion systems [28], [29].

Signal peptide search revealed that among the various ESX-1 components, the protease Rv3883c (MycP1) and the transmembrane protein Rv3882c contained N-terminal signal peptides (Table S2). Since these two proteins are not known to be secreted, it is likely that they either contribute to the mycomembrane or periplasmic component of the secretion machinery or are probably involved in the regulation of secretion (either in the periplasm-like space or in the mycomembrane).

### Prediction of glycosylation sites

Proteins with glycosylation sites are likely to be localized in the mycomembrane or periplasm-like space. Detection of such proteins may help to identify components that may contribute to the formation of the mycomembrane pore or may act as gates for the regulation of secretion. The present study predicted glycosylation sites in two ESX-1 components, namely, Rv3869 and Rv3873 (PPE68). The glycosylation of PPE68 was also experimentally observed by a previous study [31]. Interestingly, a series of glycosylation sites were predicted around residue 150 of Rv3869. This region corresponds to the long portion of this protein in the periplasm-like space (earlier described under the section ‘Prediction of transmembrane helices’), indicating that this region of the protein is likely to interact with the mycomembrane of the bacteria. The positions of the glycosylation sites for both these proteins are provided in Table S2.

### Identification of proteolytic cleavage sites

ESX-1 region is known to contain a serine protease Rv3883c (MycP1). This protease was experimentally shown to cleave one of the secreted proteins, namely Rv3881c (EspB) at at least two sites [18]. Ohol et al. (2010) identified the cleavage site to be a tetrapeptide motif with a conserved proline residue at the last position. It was observed that, more than 60% of the maximum activity of MycP1 could be retained with motifs having a profile ‘[ALV][X][ALR]P’. Thus, presence of this tetrapeptide motif in any of other ESX-1 component would probably tag that as a novel substrate of MycP1.

In the present study, a ‘LVLP’ motif was identified at the N-terminal of the ubiquitin-like domain across all orthologs of Rv3877. This suggests that, Rv3877 could be a probable substrate for MycP1. The location of the cleavage motif in Rv3877 is shown in Figure S2.

### Prediction of protein-protein association

Analysis of protein-protein associations revealed direct or indirect linkage among the ESX-1 components. This indicates that, the genes of the ESX-1 region are functionally linked to each other and probably participate in the same pathway. The analysis indicated that, most of the MCE proteins, namely Rv0170 (Mce1B), Rv0171 (Mce1C), Rv0174 (Mce1F) and Rv0177, had probable functional associations with Rv3869, Rv3870 and Rv3882c. Interestingly, these MCE proteins were also identified to have similar phylogenetic profiles as the ESX-1 components. In addition, the latter three MCE proteins (Mce1C, Mce1F and Rv0177) showed a high probability of association with few other proteins belonging to ESX-1 region (Rv3871, ESAT-6, Rv3876 and Rv3877). These results suggest that, the above proteins of MCE cluster 1 probably function together with ESX-1 components and participate in the ESX-1 secretion pathway.

### Detection of secretion islands

It is known that genes responsible for secretion of bacterial virulence factors are mostly located in genomic islands having an oligonucleotide composition distinct from the rest of the genome [32], [33]. The present study identified a compositionally distinct region spanning from Rv3874 to Rv3902c (a total of 33 genes). Interestingly, the entire ESX-1 gene cluster was observed to be located in this genomic island (Table S3). This suggests that the ESX-1 region corresponds to distinct secretion island similar to that seen in the other bacterial secretion systems. Furthermore, the same compositionally distinct region was also observed to contain ESX-2 gene cluster (Rv3884c–Rv3896c). In addition, a portion of the MCE Cluster 1 region (which also possessed similar phylogenetic profiles as ESX-1 components) was also predicted to be located in a compositionally distinct region of *M. tuberculosis* genome, spanning the genes Rv0152c to Rv0174 (Table S3).

## Discussion

The analysis of the ESX-1 region presented here in conjunction with the existing published data has been used to predict functional roles for components of the ESX-1 secretion system. Based on these insights, we propose a model for the secretion of various effector proteins by the Type VII secretion system. The proposed model provides a hypothesis based framework for the further characterization of the T7SS.

### Functional assignments of ESX-1 components

The proteins associated with the ESX-1 secretion pathway can be broadly classified into three groups – (A) secreted proteins, (B) regulatory proteins and (C) structural proteins. Details of these proteins and their probable mechanisms of action/regulation are described in the following sub-sections.

#### (A) Secreted proteins

The experimentally identified substrates of ESX-1 secretion pathway include CFP-10, ESAT-6, EspA, EspC and EspB [3], [7], [8], [14], [18], [21]. CFP-10 and ESAT-6 are known to form pairs, which adopt stable four-helix-bundle structures [8], [16], [21], [22]. Similarly, EspA and EspC have been shown to be dependent on each other and on the CFP-10/ESAT-6 pair for their stability and secretion [8], [21]. Interestingly, in the present study, the prediction of helix-turn-helix structures (for both EspA and EspC) and the detection of conserved WXG motifs linking the helices (in EspA) indicates that, EspC and EspA probably interact with each other, forming a four-helix-bundle (similar to that formed by the CFP-10/ESAT-6 pair).

EspB, another secretory protein, was previously reported to interact with Rv3879c and Rv3871 for its secretion and was also shown to regulate the secretion of CFP-10/ESAT-6 [15]. While Rv3879c was predicted to remain within the cell, EspB was shown to be secreted out. However, a separate study identified Rv3879c as a potential T-cell antigen [34]. The observation that Rv3879c is able to induce T-cell response, suggests that, Rv3879c is either secreted or is partially anchored to the mycomembrane (i.e. it undergoes at least an inner membrane translocation). Our analysis predicts both EspB and Rv3879c to contain helical structures with conserved WXG motifs (similar to CFP-10/ESAT-6), connecting the two helices. Thus, the results obtained from previous studies (EspB interacting with Rv3879c) and from our 3D fold analysis suggest that, EspB and Rv3879c probably pair up to form a four-helix-bundle like CFP-10/ESAT-6 or EspA/EspC, and cross the inner cell membrane.

Finally, the results of the 3D fold analysis seem to suggest an interesting aspect of the Type VII secretion system, which is its preference for substrates having four-helix-bundle structures. The probable reason for this is explained in later sections.

#### (B) Regulatory proteins

The results obtained from the current analysis and those known from previously reported studies have indicated a list of probable regulatory components of ESX-1 secretion pathway (Table 1). These regulatory components can be divided into two functional categories: (i) Initial activation and transcription of secreted proteins and (ii) Negative regulation of the ESX-1 secretion machinery.

**Table 1:** Functional assignments of regulatory components involved in ESX-1 secretion pathway.

Componentname	Known function (from literature)	Probable function (obtained from present study)	Function derived from
Rv3866	Cytoplasmic protein involved in expression of ESAT-6	Probably involved in transcriptional activation of ESAT-6	Domain analysis, literature
Rv3862 (WhiB6)	Transcriptional regulatory protein	Initial activation of the ESX-1 secretion machinery	Phylogenetic profile analysis, literature
Rv3876	Unknown	Negative Regulation of secretion	Domain analysis, 3D fold analysis, literature

Initial activation and transcription of secreted proteins: Our analysis showed that the phylogenetic profile of WhiB6 is identical to Rv3866 (and similar to the phylogenetic profiles of a majority of ESX-1 components). WhiB6 is a member of WhiB family of transcriptional regulators. WhiB family of proteins are unique to Actinomycetes and seven proteins belonging to the WhiB family (WhiB1-7) have been identified in M. tuberculosis H37Rv [35]. The proof for their regulatory role was established from a study where WhiB3 was shown to bind the sigma factor of RNA polymerase [36]. WhiB6 has been reported as a sensor of phagosomal signals [37]. It has also been shown to be up-regulated under several stress conditions which are typically observed inside the phagosome [35]. Interestingly, previous studies have also suggested the ESX-1 components to be involved in macrophage escape and cell to cell spread of the bacteria [7], [38]. Thus, we hypothesize that WhiB6 probably acts as a receptor of phagosomal signals and participates in initial activation of ESX-1 secretion machinery.

Our analysis identified Rv3866 to have a DNA binding domain. The protein is also reported to be involved in the synthesis and/or stability of ESAT-6 [38]. Thus, Rv3866 probably functions as a transcriptional regulator of ESAT-6. Another protein located outside ESX-1 region, PhoP (Rv0757) (not included in our study) was previously shown to regulate the operon Rv3612c-Rv3616c (including the genes corresponding to EspA and EspC), which in turn affects secretion of ESAT-6 [39].

The above observations thus suggest that, WhiB6 probably acts as an initial receptor of phagosomal signals and brings about the initial activation of the ESX-1 secretion machinery either directly or indirectly. The indirect activation probably involves other transcriptional regulators like Rv3866 and PhoP.

Negative Regulation of secretion: Our analysis identified Rv3876 to contain a FlhG domain and a three dimensional fold similar to that adopted by ParA family of proteins. The ParA family of proteins as well as proteins containing the FlhG domain are known to negatively regulate the processes of chromosome partitioning and flagellar biogenesis respectively [24], [26]. These observations suggest that, Rv3876 may have a role in negative regulation of ESX-1 region.

#### (C) Structural Proteins

The translocation process can be divided into various stages. Based on probable roles/functions derived from the present study as well as from literature, we have divided the structural components into functional categories, depending on the stages of protein translocation process to which they contribute. Details of these stages and the participating structural proteins have been discussed below and are further summarized in Table 2.

**Table 2:** Functional assignments of the structural components involved in ESX-1 secretion pathway.

Component name	Known function (from literature)	Probable function (obtained from present study)	Function derived from
**Multimeric assembly of secretory proteins**			
Rv3868	ATPase	Initiation of the assembly of multiprotein complexes	Domain analysis, 3D fold analysis, literature
**Recognition of secretory proteins and motor activity**			
Rv3870, Rv3871	Delivery of ESAT-6/CFP-10 pair to the secretion machinery	ATPase motor to recruit the secretory proteins to the inner membrane pore (Rv3877) and facilitate translocation	Domain analysis, 3D fold analysis, literature
**Inner membrane translocation**			
Rv3877	Pore forming protein located in the inner membrane	Inner membrane pore. Regulates secretion of proteins through its interaction with MycP1	Domain analysis, motif analysis, literature
**Proteolytic processing and secretion regulation at the periplasm-like space**			
Rv3883c (MycP1)	Protease which cleaves EspB (Rv3881c)	Cleaves the ubiquitin-like domain of Rv3877, thereby opening the gate and initiating translocation of secretory proteins	Motif analysis, literature
**Mycomembrane translocation**			
Rv3869	Membrane protein of unknown function	Component required for the formation of the mycomembrane translocation machinery	Domain analysis, glycosylation site prediction, protein-protein association prediction, literature
Rv3882c	Membrane protein predicted to face periplasm	Component required for the formation of the mycomembrane translocation machinery	Protein-protein association prediction, literature
MCE1 proteins (Mce1B, Mce1C, Mce1F, Rv0177)	Unknown	Component required for the formation of the mycomembrane translocation machinery	Phylogenetic profile analysis, protein-protein association prediction, literature

Multimeric assembly of secretory proteins: The TPR domain identified (in the present study) in the N-terminal region of Rv3868 suggests its probable involvement in the assembly of multi-protein complexes. This is analogous to that predicted for the protein VCA0119 in the Type VI Secretion System [40]. Furthermore, Rv3868 has also been experimentally shown to interact with CFP-10/ESAT-6 as well as the C-terminal of EspC [17], [41]. Previous studies have also provided experimental evidences that CFP-10/ESAT-6 and EspA/EspC pairs are dependent on each other for their secretion and stability [8], [21], suggesting that, the dimeric pairs of CFP-10/ESAT-6 and EspA/EspC are probably secreted as a multimeric protein assembly. Thus, the results of our analysis along with the observations of the previous studies suggest that, Rv3868 probably helps in the multimerization of CFP-10/ESAT-6/EspA/ESpC, which are subsequently secreted as a multimeric assembly. This multimeric protein assembly is probably recognized and translocated by a separate motor ATPase.

Recognition of secretory proteins and motor activity: Rv3870 is annotated as a member of Ftsk/SpoIIIE family of DNA translocase proteins [13]. It has been shown to contain two transmembrane helices and a single FtsK ATPase domain [13]. It is known to interact with another cytoplasmic FtsK/SpoIIIE family protein Rv3871 [3], [13], containing two FtsK ATPase domains. Earlier studies also provided evidence that, Rv3871 interacts with the C-terminal region of CFP-10, and recruits the CFP-10/ESAT-6 pair to the translocon unit [3], [7], [19], [20]. The α and β units of the FtsK domain containing proteins are known to multimerize to produce a hexameric ring [42], with an inner diameter of ∼30 Å. This ring then acts as a DNA pump which translocates double stranded DNA during cell division [42]. Since Rv3870 contains one FtsK domain (one pair of α and β domain) and Rv3871 contains two such domains (two pairs of α and β domains), we propose that, the α and β domains contributed by two Rv3870/Rv3871 pairs could form a hexameric ring, similar to that formed by a hexamer of FtsK proteins.

Moreover, we performed an estimation of the approximate diameters (summarized in Text S1) of four-helix-bundle structures (corresponding to the CFP-10/ESAT-6 structural homologues). Our structural analysis indicates that, the approximate diameter of such four-helix-bundle structures is between 20–23 Å. This diameter is similar to that of double stranded DNA molecules, which is observed to be around 20 Å [43], [44]. These observations indicate that, the FtsK family proteins (Rv3870 and Rv3871) probably recruit and translocate the (four-helix-bundle) substrates in a manner similar to that used by FtsK for the translocation of double stranded DNA. Thus, the proteins Rv3870/Rv3871 of T7SS may provide a unique example wherein, a machinery initially believed to be used for translocation of double stranded DNA substrates, has been tuned to transport protein complexes. A similar component related to FtsK family, VirD4, has been shown to participate in the Type IV secretion system [28], which is responsible for translocation of both DNA as well as protein moieties.

Inner membrane translocation: The subsequent step after recognition and recruitment of the secretory proteins, is movement of these proteins through the inner membrane pore. Rv3877 has previously been suggested to be a pore forming protein through which the secretory proteins cross the inner membrane [4]. As an additional analysis, we have determined the diameter of the pore probably formed by the TM helices of Rv3877. Considering a compact arrangement of the helices, the diameter of the pore was determined to be approximately 26 Å (Text S2). The above calculation assumes a tight packing among the constituent helices (ignoring the side chain lengths). Thus, the actual diameter is likely to increase upon incorporating the side-chain lengths and interactions. This suggests that, the pore formed by Rv3877 will probably be able to translocate the substrate pairs (in the form of four-helix-bundles) of the ESX-1 secretion pathway.

Proteolytic processing and secretion regulation at the periplasm-like space: Our analysis identified MycP1 to be located in the periplasm-like space probably anchored to the inner membrane by a C-terminal transmembrane helix. The cleavage of EspB by MycP1 was shown to be not only essential for its secretion, but also necessary for the controlled secretion of CFP-10/ESAT-6 [18]. While the knock-out of MycP1 was observed to completely abolish secretion, the presence of a mutant MycP1 without any protease activity was shown to increase the secretion of CFP-10/ESAT-6 and accumulation of EspB in the periplasm-like space [18]. These observations indicate a probable regulatory role of this serine protease (MycP1) in the periplasm-like space. Furthermore, in our analysis, the identified MycP1 cleavage motif within the N-terminal ubiquitin-like domain of Rv3877 indicates that, the N-terminal domain of Rv3877 probably acts as a gate, the cleavage of which by MycP1 may be necessary for the opening of the inner membrane channel and initiating translocation.

Mycomembrane translocation: The subsequent step in the secretion mechanism is the translocation of proteins through the mycomembrane. Currently, the exact components of the ESX-1 secretion machinery involved in the transport across mycomembrane have not been identified. Only three outer membrane pore proteins (OmpA, Rv1698 and Rv1973) have been experimentally characterized in mycobacterial species and none of them is reported to be linked to the ESX-1 secretion pathway [45]–[47]. Thus, one of the goals of our analysis was to identify probable candidates that could aid in the transport of substrates across the mycomembrane.

The hypothetical protein, Rv3869 has been previously shown to be an essential component of the ESX-1 secretion machinery, as disruption of this gene was observed to result in the loss of CFP-10/ESAT-6 secretion [22]. Our analysis predicted that both Rv3869 and Rv3882c contain long C terminal domains protruding into the periplasm-like space. These observations, along with the detection of a series of glycosylation sites in this region of Rv3869 suggests the probable association of both Rv3869 and Rv3882c with the periplasm-like space and/or the mycomembrane. These results also corroborate with those obtained by Mah et al. (2010), which considered several other properties like presence of signal peptides, beta content, etc. and predicted both these proteins as outer (or myco) membrane pore forming proteins (OMP). Based on these observations, we hypothesize that, Rv3869 and Rv3882c could act as probable mycomembrane translocon components of the ESX-1 secretion machinery, similar to the outer membrane components of other secretion systems.

Other than Rv3869 and Rv3882c, our analysis (using phylogenetic profiles as well as protein-protein association networks) suggests four proteins (Mce1B, Mce1C, Mce1F and Rv0177) corresponding to MCE cluster 1 to have probable functional linkage to the ESX-1 secretion pathway. Additionally, these proteins were also predicted earlier as probable outer membrane pore forming proteins [45], [46], [48]. Thus, we hypothesize that, these proteins could act as accessory components of the ESX-1 secretion machinery, to aid in the transport of secretory proteins across the mycomembrane.

Our analysis thus suggests that the probable candidates which may constitute the mycomembrane transolocon machinery are Rv3869, Rv3882c, Mce1B, Mce1C, Mce1F and Rv0177. These proteins, either independently or in some combination, probably form the mycomembrane translocon machinery of the ESX-1 secretion system.

Additional components: Apart from the components described above, genes encoding two additional proteins of PE and PPE family (PE35 and PPE68) are located inside the ESX-1 gene cluster. While PE35 has been reported to be secreted [8], no such data is available for PPE68. PE35 has also been shown to affect the expression of CFP-10/ESAT-6 [22]. An experimental study reported PPE68 as a cell envelope associated protein [10]. Similarly, another study suggested PPE68 to function as a gating component for the controlled secretion of effector molecules [38]. These results corroborate with the results from our analysis which predicts PPE68 to be associated with the mycomembrane and/or the periplasm-like space (based on the series of glycosylation sites predicted in this protein).

The PE/PPE pair was earlier shown to adopt a four-helix-bundle structure with one helix-turn-helix being contributed by the PE protein and the other contributed by the PPE protein [27]. The tip of the structure (formed solely by the PPE counterpart) contains an elongated solvent accessible hydrophobic patch which can provide a probable interaction site for proteins containing similar patches. These observations suggest that the PE/PPE pair is likely to regulate the secretion of effector molecules by interacting with the ESX-1 components forming the mycomembrane translocon.

### Proposed model of Type VII secretion system

**Figure 2:**
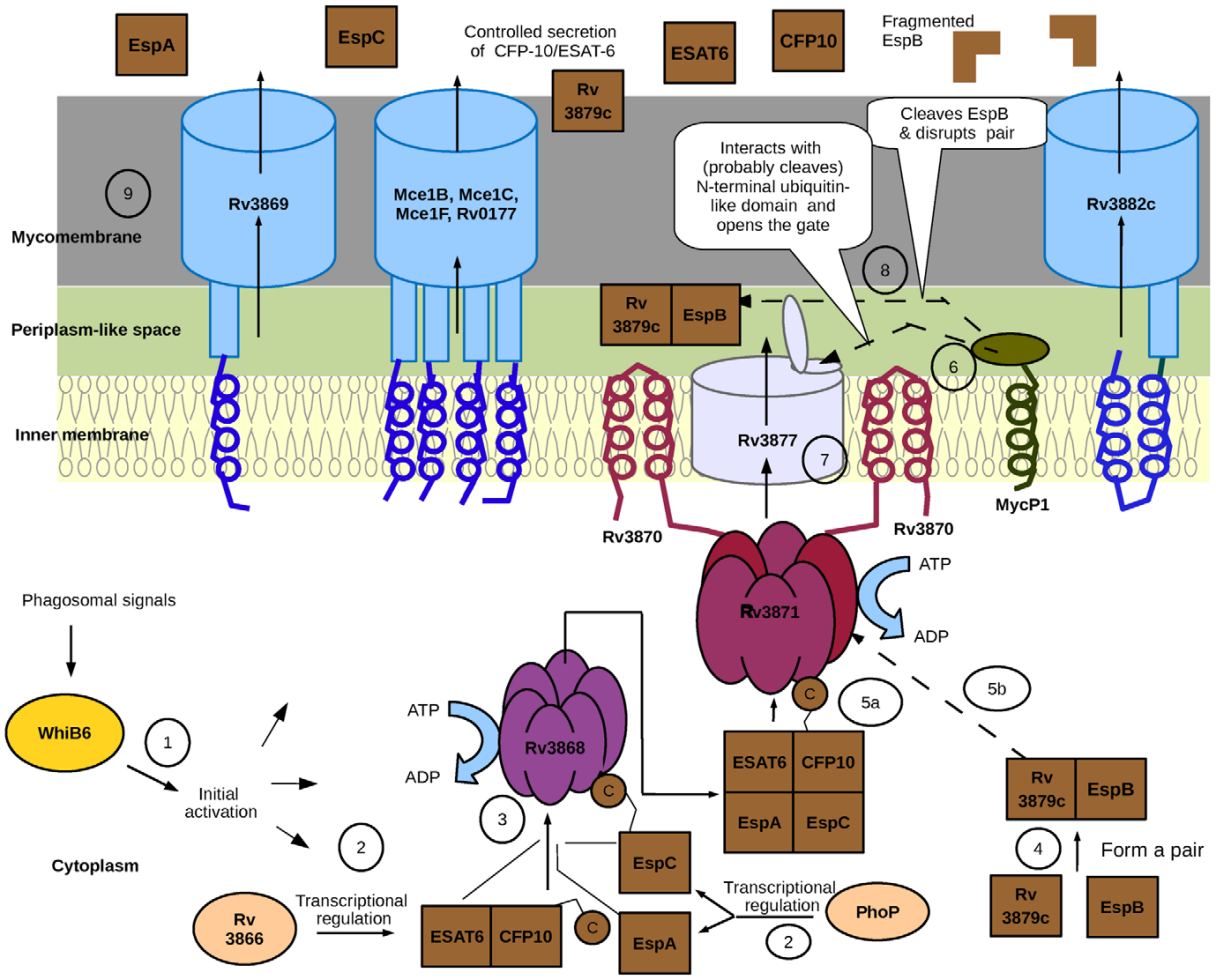
Proposed model for secretion of substrates (CFP-10, ESAT-6, EspA, EspC, EspB) using Type VII secretion system. The various steps involved are indicated by numbers and are described below: (1) Initial activation by WhiB6; (2) Transcriptional regulation of the secretory proteins by Rv3866 and PhoP; (3) Multimeric assembly of secretory proteins by Rv3868; (4) Pairing up of secretory protein EspB with Rv3879c; (5a) Recruitment of CFP-10/ESAT-6/EspA/EspC multimer by Rv3870 and Rv3871 to the inner membrane pore (Rv3877); (5b) Recruitment of EspB/Rv3879c pair by Rv3870 and Rv3871 to the inner membrane pore (Rv3877); (6) Interaction of MycP1 with Rv3877; (7) Inner membrane translocation of secretory proteins; (8) Cleavage of EspB by MycP1; (9) Mycomembrane translocation of secretory proteins. The dashed arrows show interaction between the components connected by the arrow.

Combining the results of our analysis with those known from literature, we propose the following model for secretion of proteins through ESX-1 secretion machinery. The overall model is depicted in Figure 2.

### Secretion of CFP-10, ESAT-6, EspA and EspC

The initiation event for secretion of virulence factors through ESX-1 secretion pathway may depend on phagosomal signals, which are sensed by WhiB6. The WhiB6 in turn, may directly or indirectly bring about the transcriptional initiation of the machinery. Subsequently, PhoP and Rv3866 facilitate the expression of the pairs EspA/EspC and CFP-10/ESAT-6, respectively. Once the two pairs (CFP-10/ESAT-6 and EspA/EspC) are expressed, Rv3868 interacts with both the pairs and the TPR domain of Rv3868 helps in assembly of CFP-10/ESAT-6/EspA/EspC into a multimer. Rv3871, a FtsK family protein, then recognizes the multimeric protein assembly of CFP-10/ESAT-6/EspA/EspC through its specific interaction with the C-terminal end of CFP-10. Rv3871 interacts with another FtsK family protein Rv3870 and forms a translocase pore (as observed for other Ftsk family of proteins). Once recognized by Rv3871, the multimeric protein assembly (CFP-10/ESAT-6/EspA/EspC) is likely to pass through this translocase pore. This translocase pore not only recruits the substrates to the inner membrane pore protein (Rv3877), but also facilitates the ATP-dependent translocation of the substrates through the inner membrane. The interaction (and possibly the cleavage) of the N-terminal gate-like domain of Rv3877 by MycP1 opens the pore, thus enabling translocation of the substrate assembly from cytoplasm to the periplasm-like space. Subsequently, the substrate assembly crosses the mycomembrane through a channel formed by Rv3869, Rv3882c and the four proteins of MCE cluster 1 (Mce1B, Mce1C, Mce1F and Rv0177), either exclusively or in a collaborative manner.

### Secretion of EspB

EspB pairs up with Rv3879c in a manner similar to the two pairs of secretory proteins namely, CFP-10/ESAT-6 and EspA/EspC. Adopting a strategy similar to that described in the previous section, Rv3871 then recognizes and recruits this pair to the inner membrane pore formed by Rv3877. Using this pore, the EspB/Rv3879c pair moves to the periplasm-like space in a manner similar to that of CFP-10/ESAT-6/EspA/EspC. Subsequently, MycP1 cleaves EspB in the periplasm-like space, which disrupts the EspB/Rv3879c pair, resulting in the secretion of EspB. The translocation of EspB through the mycomembrane probably involves the same machinery used by the other secretory proteins (CFP-10/ESAT-6 and EspA/EspC).

### Regulation of protein secretion

The cleavage of EspB by MycP1 leads to a controlled secretion of both the CFP-10/ESAT-6/EspA/EspC multimer and EspB. The controlled secretion of these proteins is probably achieved due to their competition for translocation (through the mycomembrane) using the same machinery. However, the increase in secretion of CFP-10/ESAT-6 despite the absence of the protease activity of MycP1 as observed by Ohol et al. (2010) is interesting and the molecular mechanism governing this phenomena needs to be resolved experimentally in the laboratory. On the other hand, in the absence of MycP1, neither the cleavage of ubiquitin-like domain of Rv3877 nor its interaction with MycP1 occurs. Thus, the gate remains closed resulting in the loss of secretion of all secretory proteins.

### Conclusion

The present study, for the first time, suggests probable functional roles for some of the previously uncharacterized components of ESX-1 secretion system, namely Rv3866, Rv3876, Rv3869 and Rv3882c. While Rv3866 has been proposed to be involved in the transcriptional activation of the secretion machinery, Rv3876 has been predicted to be a negative regulator of this machinery. On the other hand, Rv3869 and Rv3882c have been identified as probable components of the mycomembrane translocation apparatus. The present analysis also proposes a few components that were not known to be associated with the ESX-1 secretion system. These include an additional transcriptional regulator (WhiB6) and components associated with transport through the mycomembrane (MCE Cluster 1 proteins).

With regards to the secretory proteins of the ESX-1 secretion pathway, the current analysis indicates that the secretory protein pairs have a preference to adopt a four-helix-bundle structure. While the CFP-10/ESAT-6 pair was previously reported to have such a structure, the present analysis suggests that the other secretory protein pairs (EspA/EspC and EspB/Rv3879c) probably also adopt similar structures.

One of the crucial aspects of the ESX-1 secretion mechanism pertains to the translocation of secretory proteins through the mycomembrane. In spite of considerable experimental efforts to understand the ESX-1 secretion system, the components involved in protein translocation through the mycomembrane is currently not known. The identification of the probable candidates (Rv3869, Rv3882c and the MCE Cluster 1 proteins) constituting the mycomembrane translocation apparatus is thus one of the most important outcomes from the current analysis.

Thus, combining the results of the present computational analysis with those known from literature, the current study suggests a much more comprehensive model of ESX-1 secretion system.

## Methods

**Figure 3:**
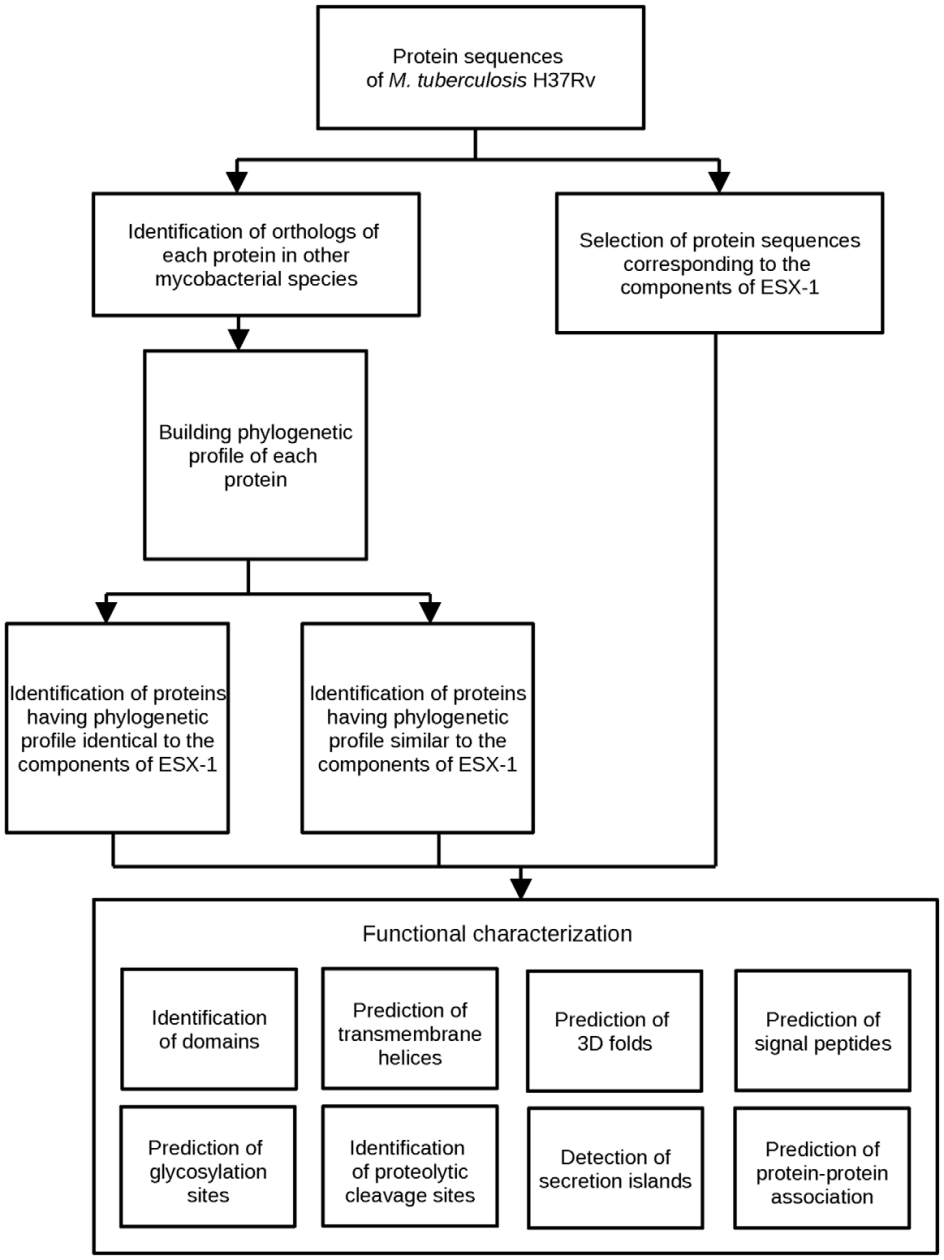
Workflow showing various methodologies used for functional annotation of components of Type VII secretion system in *M. Tuberculosis*.

Protein sequences belonging to 14 completely sequenced mycobacterial species were downloaded from the NCBI database (http://www.ncbi.nlm.nih.gov/) (Table S1). These species belonged to diverse clades of the *Mycobacterium* genus. The protein sequences corresponding to the 17 gene components from *M. tuberculosis* H37Rv (Table S4), earlier identified experimentally to be involved in ESX-1 secretion pathway [5], [7], [22], [38], were selected and subjected to various in silico analysis. Figure 3 describes the schematic workflow of various bioinformatics analysis performed on these proteins.

### Orthology search and phylogenetic profile

In order to identify the pattern of conservation of the genes involved in ESX-1 secretion pathway, a phylogenetic profile based approach was adopted. First, orthologs of each of the 3989 proteins of *M. tuberculosis* H37Rv were identified in 13 other mycobacteria (taking one representative strain from each species) using a standalone version of the BLASTp program [49]. The orthology search was restricted to only mycobacteria since organisms outside the *Mycobacterium* genus mostly possess orthologs of the genes belonging to only ESX-4 [4]. For each protein, only those hits which had an e-value of less than e^−4^ and alignment coverage of at least 60% of the length between the query and the subject proteins (in the BLASTp alignment) were retained as significant hits. This was done to ensure that the identified list of probable orthologous hits (for a given protein) retained the domain architectures of the query protein. Finally, the true ortholog for each protein was identified using the reverse BLAST approach [23].

A phylogenetic profile matrix was generated with all proteins of *M. tuberculosis* H37Rv. The matrix had 3989 rows and 14 columns, where rows represented proteins of *M. tuberculosis* H37Rv and columns represented the 14 mycobacterial species. A given element H_ij_ in this matrix represented information about the presence/absence of the ortholog of an *M. tuberculosis* H37Rv protein ‘i’ in mycobacterial species ‘j’. Thus, 0 and 1 in the matrix corresponded to absence and presence of orthologs, respectively. It is to be noted that the presence or absence (of orthologs) have been termed with respect to the true orthologs (identified after the reverse BLAST step).

Subsequently, proteins with identical profiles were clustered together. A subset of these clusters, containing one or more ESX-1 components, were identified for further analysis. All proteins having similar profiles to these clusters (defined as the neighbourhood space) were then obtained, since they are expected to have functional linkage with the ESX-1 components (present in these clusters). Here, the similarity of phylogenetic profiles was quantified in terms of the bit difference as defined by Pellegrini et al. (1999). In the present analysis, all proteins having a bit difference of less than or equal to three to a given cluster (containing ESX-1 components) were identified to have a similar phylogenetic profile.

### Domain search

In order to detect the presence of known functional domains, protein sequences corresponding to each of the 17 ESX-1 components of *M. tuberculosis* were queried against various domain databases. The databases used for domain search were PRODOM (http://prodom.prabi.fr/prodom/current/html/form.php) [50], CDD (http://www.ncbi.nlm.nih.gov/Structure/cdd/wrpsb.cgi) [51] and Interpro (http://www.ebi.ac.uk/Tools/InterProScan/) [52].

### Prediction of transmembrane helices

To detect the presence of transmembrane regions, protein sequences of each of the 17 ESX-1 components and the probable interacting proteins were submitted to three transmembrane prediction servers, namely, TMHMM (http://www.cbs.dtu.dk/services/TMHMM/) [53], TMPRED (http://www.ch.embnet.org/software/TMPRED_form.html) [54] and SOSUI (http://bp.nuap.nagoya-u.ac.jp/sosui/sosui_submit.html) [55]. Proteins predicted to have the transmembrane regions by at least two of these methods were identified.

### Prediction of 3D folds

The HHPred web server (http://toolkit.Tuebingen.mpg.de/hhpred/) [56] was used to predict the 3D folds in all the 17 ESX-1 components of *M. tuberculosis*. This tool predicts 3D fold based on pairwise comparison of profile hidden Markov models (HMMs).

### Signal peptide search

In order to identify potential candidates forming the mycomembrane component of the ESX-1 secretion machinery in *M. tuberculosis*, a tool called SignalP (http://www.cbs.dtu.dk/services/SignalP/) [57] was used to detect the presence of N-terminal signal sequences in the analyzed set of proteins.

### Prediction of glycosylation sites

Besides searching for the presence of N-terminal signal peptides, the protein sequences under study were also submitted to the NetOglyc web server (http://www.cbs.dtu.dk/services/NetOGlyc/) [58] for the detection of putative structural components localizing to the mycomembrane or periplasm-like space. The NetOGlyc server performs Neural Network based prediction of glycosylation sites in human protein sequences. However, it has been previously demonstrated that it can accurately predict the presence of glycosylation sites in mycobacterial proteins [59]. In order to increase the reliability of this analysis, the results generated by the tool were further filtered by considering only those proteins which had at least three glycosylation sites within a ten amino acid window.

### Identification of proteolytic cleavage sites

Multiple sequence alignments for the orthologs of all ESX-1 components of *M. tuberculosis* were performed using ClustalW (http://www.ebi.ac.uk/Tools/clustalw/) [60]. Subsequently these alignments were searched for the presence of the tetrapeptide motif having the MycP1 cleavage signature ‘[ALV][X][ALR]P’ (as observed by Ohol et al. 2010).

### Prediction of protein-protein association

Probable protein-protein associations were predicted using the MtbPPI tool (http://www.cdfd.org.in/MtbPPI/). The tool contains a comprehensive protein linkage network of *M. tuberculosis* H37Rv, predicted using Support Vector Machine with parameters derived from various genomic context methods (phylogenetic profile, gene distance, etc.) and microarray expression data. The network captures all known interacting pairs in *M. tuberculosis*. Thus, the association among the proteins involved in ESX-1 secretion can be verified using this network.

### Detection of secretion islands

The components of Type I–IV and Type VI secretion systems were previously found to be located in compositionally distinct genomic regions [32], [33]. In order to check whether the ESX-1 region occurs in compositionally distinct islands, *M. tuberculosis* H37Rv genome was given as input to the INDeGenIUS program [61]. The program identifies regions in a genome possessing a deviant oligonucleotide composition as compared to the rest of the genome.

## Supporting Information

Figure S1 **Predicted 3D folds and their location in seven ESX-1 components.**
(PDF)

Figure S2 **Conserved motifs in some of the ESX-1 secreted proteins and components identified using multiple sequence alignments of the corresponding orthologs.** These include the conserved WXG motif in (A) Rv3881c (EspB), (B) Rv3879c, (C) Rv3616c (EspA) and (D) conserved cleavage motif for Rv3883c (MycP1) in Rv3877.
(PDF)

Text S1 **Approximation of the diameter of the four helix bundle formed by the CFP-10/ESAT-6 pair and its homologous structures available in the Protein Data Bank (PDB).**
(PDF)

Text S2 **Calculation of inner diameter of the cytoplasmic membrane pore Rv3877.**
(PDF)

Table S1 **List of mycobacterial species and the number of orthologs of the ESX-1 components.**
(PDF)

Table S2 **Summary of the predicted domains, transmembrane helices, 3D folds, presence of signal peptides and glycosylation sites in ESX-1 components.**
(PDF)

Table S3 **Genes constituting the compositionally distinct islands that harbor (A) ESX-1 gene cluster and, (B) a part of the MCE Cluster 1 region.**
(PDF)

Table S4 **List of gene components experimentally identified to be involved in ESX-1 secretion pathway.** The genes and the corresponding protein names are taken from the TubercuList database (http://tuberculist.epf1.ch/).
(PDF)
