# Supplementary material for: Computational Analysis of the ESX-1 Region of Mycobacterium tuberculosis: Insights into the Mechanism of Type VII Secretion System
Source: PLoS One. 2011 Nov 30;6(11):e27980. doi: 10.1371/journal.pone.0027980 (PMC3227618; doi:10.1371/journal.pone.0027980)

Figure S1: Predicted 3D folds and their location in seven ESX-1 components.

(1) Rv3868

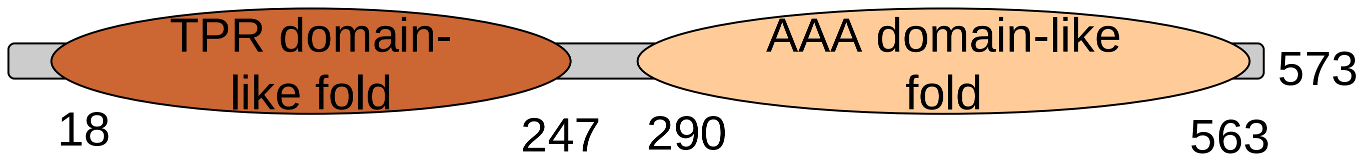

(2) Rv3876

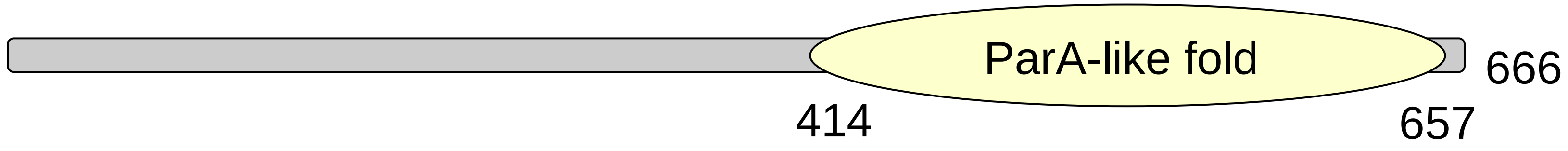

(3) Rv3877

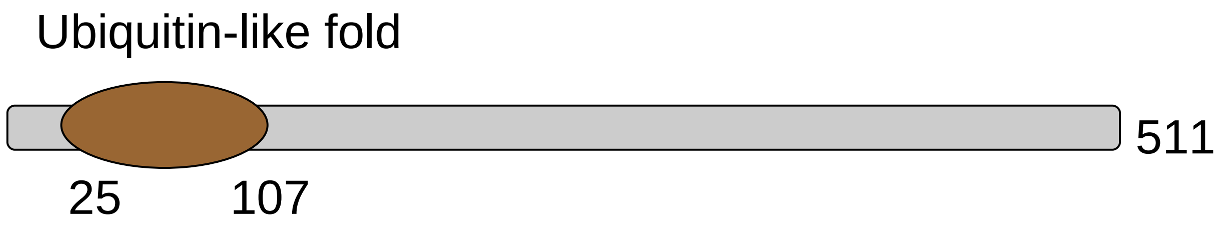

(4) Rv3881c (EspB)

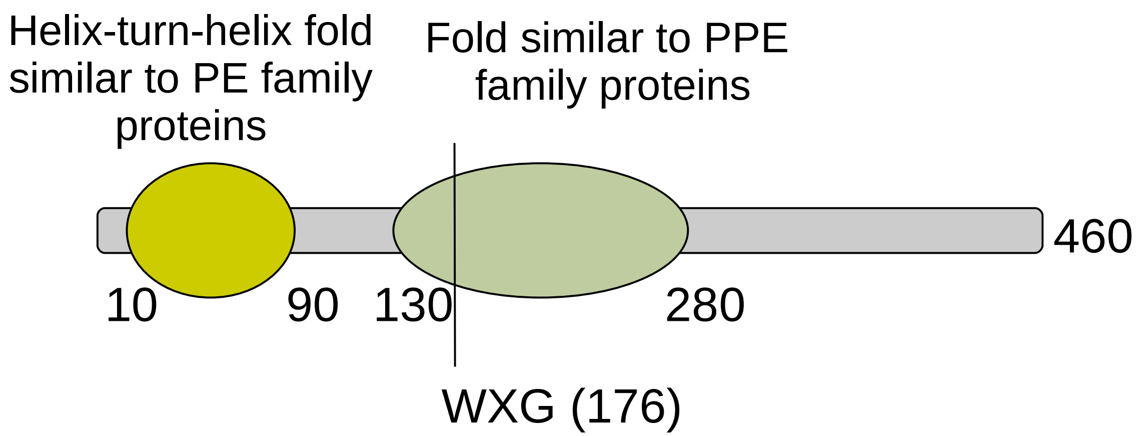

(5) Rv3879c

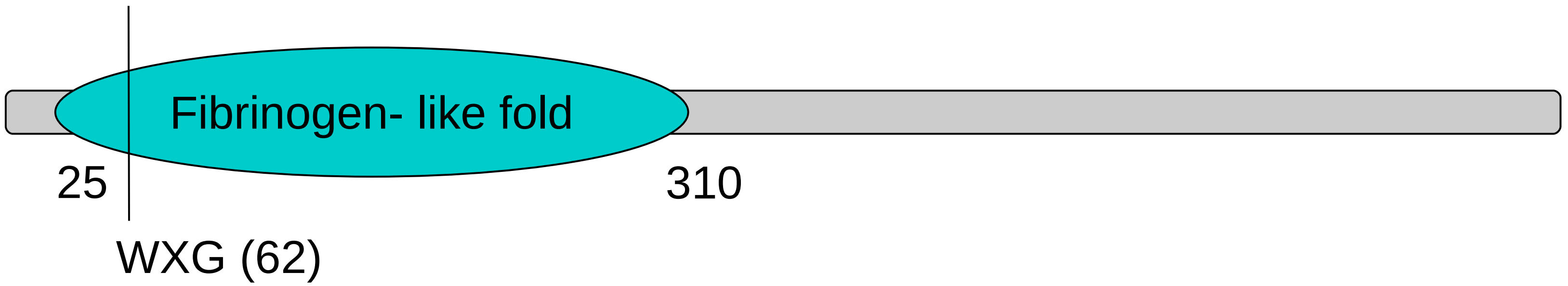

(6) Rv3615c (EspC)

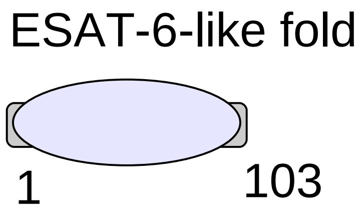

(7) Rv3616c (EspA)

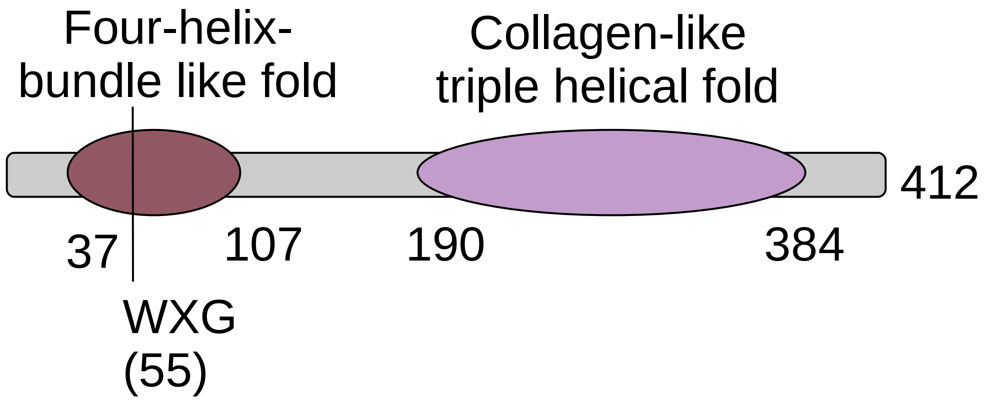

Supplement: Figure S1 — Predicted 3D folds and their location in seven ESX-1 components. (PDF) [file pone.0027980.s001.pdf]
