# Supplementary material for: Computational Analysis of the ESX-1 Region of Mycobacterium tuberculosis: Insights into the Mechanism of Type VII Secretion System
Source: PLoS One. 2011 Nov 30;6(11):e27980. doi: 10.1371/journal.pone.0027980 (PMC3227618; doi:10.1371/journal.pone.0027980)
Supplement: Figure S2 — Conserved motifs in some of the ESX-1 secreted proteins and components identified using multiple sequence alignments of the corresponding orthologs. These include the conserved WXG motif in (A) Rv3881c (EspB), (B) Rv3879c, (C) Rv3616c (EspA) and (D) conserved cleavage motif for Rv3883c (MycP1) in Rv3877. (PDF) [file pone.0027980.s002.pdf]

Figure S2: Conserved WXG motif in (A) Rv3881c (EspB), (B) Rv3879c, (C) Rv3616c (EspA) and (D) conserved cleavage motif for Rv3883c (MycP1) in Rv3877, identified using multiple sequence alignments of the corresponding orthologs.

(A)

```
gi|15611017|[tuberculosis]       T-GDQGASLAHFADGWNTFNLTLQGDVKRFRG-FDNWEGDAATACEASLD 189
gi|31795055|[bovis]              T-GDQGASLAHFADGWNTFNLTLQGDVKRFRG-FDNWEGDAATACEASLD 189
gi|240168361|[kansasii]          S-GDQGRSLVDFANAWNDYNFALQGDVKRFRA-FDNWEGDAATACEASLD 192
gi|183984318|[marinum]           NKGDQGKSLLHFADEWTTYAQTIRDSFSRFRE-FEDWDGDAAETVQDALL 190
gi|118618031|[ulcerans]          W-ADNGNSFENFARTWLDYRLKLLEATDRFGP-FQRWDGEAAAAVEDHFD 192
gi|145221352|[gilvum]            E-GDQGASLRAAATEWTANAARLTEAAIPFEVRMQNWEGVAAEAAYTKLK 197
                                   .*:* *:   *  *      :      *   :: *:* ** :    :
```

(B)

```
gi|240170251|[kansasii]          EQRLHVFEGGVWSGGAANAANDGLGANIDQLVMLQHHLATVITWHRDIAD 100
gi|183984322|[marinum]           QQRLHVFEGGVWSGGAANAANGALGANIDQLMTVQDHLATVIAWHRYVAD 100
gi|15611015|[tuberculosis]       QQKGHVFEGGLWSGGAANAANGALGANINQLMTLQDYLATVITWHRHIAG 100
gi|31795053|[bovis]              QQKGHVFEGGLWSGGAANAANGALGANINQLMTLQDYLATVITWHRHIAG 100
                                 :*: ******:**********..*****:**: :*.:*****:*** :*.
```

(C)

```
gi|15610752|[tuberculosis]       PGDGWLGSAADKYAGKNRNHVNFFQELADLDRQLISLIHDQANAVQTTRD 100
gi|121639535|[bovis BCG]         PGDGWLGSAADKYAGKNRNHVNFFQELADLDRQLISLIHDQANAVQTTRD 100
gi|240173316|[kansasii]          PGDSWQGSAAENYAGKNKDQLNFFQQLAELDRELQQLIADQANAVEKTRD 100
gi|183984137|[marinum]           PGDGWLGSAADHYSGKNRKHVNFFKELAELDRELQKLIADQASAVKTTRE 100
gi|15827124|[leprae]             PGDGWLGSAADKYAGQNRKRVDIFQELAELDKELIELIHNQANSVQTTRG 100
                                 ***.* ****::*:*:*:.::::*::**:**::* .** :**.:*:.**
```

(D)

```
gi|108797054|[MCS]               --MTTTAAASTTSSVTPGRPSTTRVTILTGRRMTDLVLPAAAPVETYIDE 48
gi|119866139|[KMS]               ------------------------MTILTGRRMTDLVLPAAAPVETYIDE 26
gi|126432676|[JLS]               ------------------------MTILTGRRMTDLVLPAAAPVETYIDE 26
gi|118473897|[smegmatis]         --MTSTAAAADTSSVTPGRPSTTRVTILTGKRMTDLVLPATAPIETYIDE 48
gi|120401109|[vanbaalenii]       --MTATVAPPTSSNVTPGRPTTTRVTILTGRRMTDLVLPSAAPIETYVDE 48
gi|145221359|[gilvum]            ------------------------MTILTGRRMTDLVLPSAAPIETYVDE 26
Rv3877|[tuberculosis]            MSAPAVAAGPTAAGATAARPATTRVTILTGRRMTDLVLPAAVPMETYIDD 50
gi|31795051[bovis]               MSAPAVAAGPTAAGATAARPATTRVTILTGRRMTDLVLPAAVPMETYIDD 50
gi|183178749|[marinum]           --------------------------------MTDLVLPAAAPMESYVDE 18
gi|15826897|[leprae]             MSAPAVTAGPATAGITPARPSATRVTILTGKRMTDLVLPSTVSIEAYIDE 50
                                                                 *******::..:*:*:*:
```
