# Supplementary material for: Computational Analysis of the ESX-1 Region of Mycobacterium tuberculosis: Insights into the Mechanism of Type VII Secretion System
Source: PLoS One. 2011 Nov 30;6(11):e27980. doi: 10.1371/journal.pone.0027980 (PMC3227618; doi:10.1371/journal.pone.0027980)
Supplement: Text S1 — Approximation of the diameter of the four helix bundle formed by the CFP-10/ESAT-6 pair and its homologous structures available in the Protein Data Bank (PDB). (PDF) [file pone.0027980.s003.pdf]

Text S1: Approximation of the diameter of the four helix bundle formed by the CFP-10/ESAT-6 pair and its homologous structures available in the Protein Data Bank (PDB)

The document describes the methodology adopted for determining the approximate diameters of the four helix bundle structures adopted by the CFP-10/ESAT-6 pair and its homologous structures.

Table A: PDB Ids of the four helix bundle structures of the CFP-10/ESAT-6 and other homologous pairs considered for the analysis.

**Organism**	**PDB of CFP-10/ESAT-6 (homologous) structure**
Mycobacterium tuberculosis	1WA8 (CFP-10/ESAT-6 pair)
	3O9O
	3FAV
Staphylococcus	2VRZ
	2VSO
Streptococcus	3GVM
	3GWK

Step1: Determination of the helix boundaries

For each four helix bundle structure, the boundaries of the constituent helices were determined by manual inspection using RASMOL. The boundaries determined for each structure are given in Table B.

Table B: Residues constituting the alpha helices of the four-helix-bundle structures included in the analysis.

**PDB ID**	**Helix 1**	**Helix 2**	**Helix 3**	**Helix 4**
1WA8	7-39 of chain A	46-78 of chain A	608 – 640 of chain B	649 – 679 of chain B
3090	7-42 of chain A	48-95 of chain A	7-42 of chain B	48-94 of chain B
3FAV	11-38 of chain A	44-84 of chain A	10-44 of chain B	48-81 of chain B
2VRZ	7-43 of chain A	45-93 of chain A	7-43 of chain B	48-87 of chain B
2VSO	7-43 of chain A	48-85 of chain A	7-42 of chain B	48-86 of chain B
3GVM	8-43 of chain A	49-95 of chain A	8-44 of chain B	49-97 of chain B
3GWK	8-44 of chain C	49-95 of chain C	8-44 of chain E	49-97 of chain E

Step2: Determination of approximate outer diameters of the four helix bundles

For a given PDB structure, once the boundaries of the constituent helices are identified, the following steps are adopted for each residue R1 lying within the helical boundaries. The steps are also illustrated in Figure A.

1. Determination of the four helix bundle diameter at a given position (corresponding to a given residue).

a. Identification of the closest neighbors (corresponding to the given residue) in the neighboring helices:

For a given residue R1, the closest neighbor in a neighboring helix H1 is first identified by computing the CA-CA distance between R1 and all the residues constituting the neighboring helix H1. Subsequently the residue in H1 that has the minimum CA-CA distance with R1 is selected as the closest neighbor for R1. In a similar manner, the closest neighbors of R1 in each of the three interacting helices in the bundle is determined.

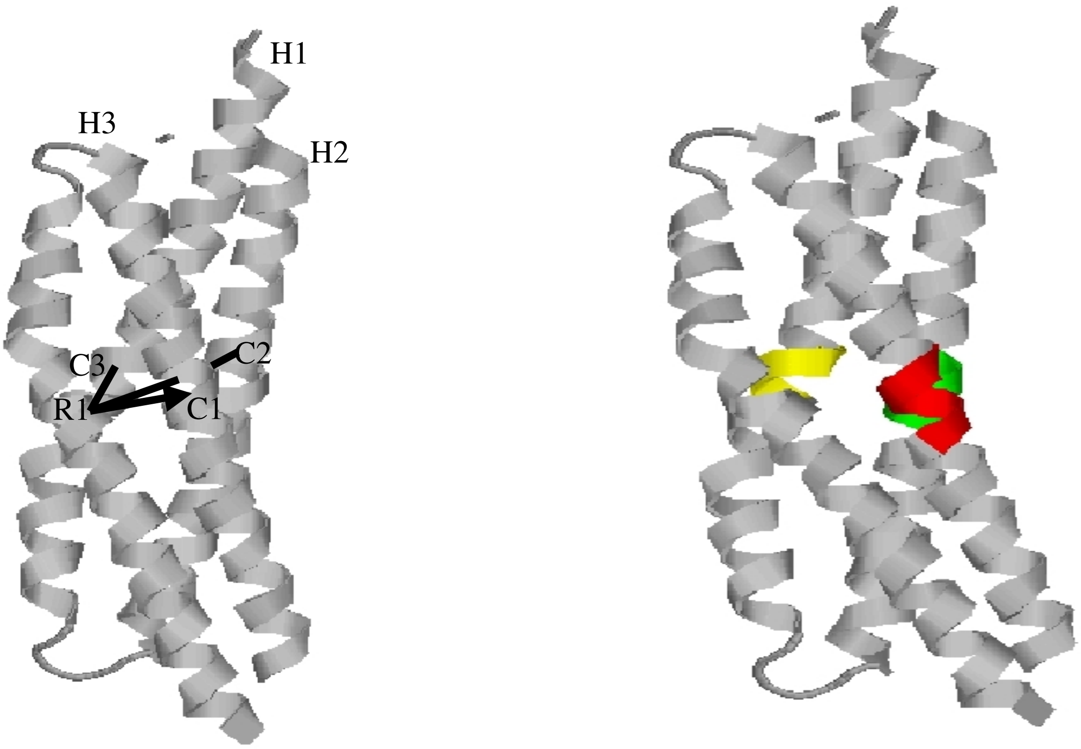

Figure A: (i)Identification of closest neighbors C1, C2 and C3 of R1 in the corresponding neighboring helices H1, H2 and H3 respectively. (ii) Identification of the neighborhood list for R1. The neighborhood list of R1 consists of all residues at positions C1, C2, C3 as well as those at positions -1, -2, +1 and +2 corresponding to C1, C2 and C3. The neighborhood lists for R1 in the helices H1, H2 and H3 are shown in red, green and yellow respectively.

The closest neighbors as well as the residues at positions i-2, i-1, i+1 and i+2 (encompassing a full turn) corresponding to each closest neighbor are identified and added to a neighborhood list N.

b. Computation of pairwise distances between the atoms of R1 and the atoms belonging to the residues constituting the neighborhood list N.

Subsequently, pairwise distances are computed between R1 and the atoms belonging to the residues constituting the neighborhood list N. The maximum distance obtained in this step gives an approximate measure of the diameter of the four helix at residue position R1.

2. Determination of average and median of the diameters of the four helix bundle

In a similar manner, the approximate four helix bundle diameter is obtained for all the residue positions constituting the helices. The average of all the values obtained for each four helix bundle structure is given in Table C.

Table C: Four helix bundle structures of the CFP-10/ESAT-6 and other homologous pairs considered for the analysis along with the average diameters of the four-helix-bundles.

Organism	PDB ID	Diameter(Å)
Mycobacterium tuberculosis	1WA8	20.25
	3O9O	22.72
	3FAV	20.83
Streptococcus	3GVM	22.72
	3GWK	22.72
Staphylococcus	2VRZ	21.77
	2VSO	21.27
