# Supplementary material for: Computational Analysis of the ESX-1 Region of Mycobacterium tuberculosis: Insights into the Mechanism of Type VII Secretion System
Source: PLoS One. 2011 Nov 30;6(11):e27980. doi: 10.1371/journal.pone.0027980 (PMC3227618; doi:10.1371/journal.pone.0027980)
Supplement: Text S2 — Calculation of inner diameter of the cytoplasmic membrane pore Rv3877. (PDF) [file pone.0027980.s004.pdf]

Text S2: Calculation of inner diameter of the cytoplasmic membrane pore Rv3877.

Rv3877 possess 10 or 11 transmembrane (TM) helices. As the structure is unknown, while calculating the diameter of the pore formed by Rv3877, the most compact organization of 10 TM helices were considered, where the 10 helices were arranged one after another forming a compact circle. Figure A shows a schematic diagram of the arrangement of 10 TM helices. The diameter of each helix being 12 Å, the inner diameter of the pore was obtained around 26 Å. The procedure is described below.

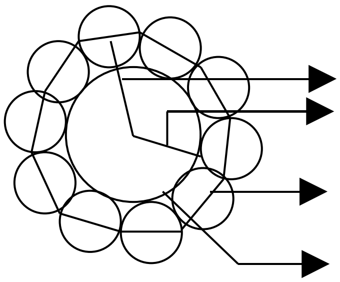

Figure A: Compact arrangement of 10 Tm helices.

The inner diameter of the pore can be obtained from the expression mentioned below.

$$2\Pi(r+6)=10*\mathit{diameter\ of\ one\ TM\ helix} \qquad \mathit{where}, r=\mathit{Radius\ of\ the\ pore}$$

$$r+6=(10*12)/2\mathit{\Pi}$$

$$r=13.1\mathit{Å}$$

$$2\mathrm{r}=26.2\mathit{Å}$$
