# Supplementary material for: Computational Analysis of the ESX-1 Region of Mycobacterium tuberculosis: Insights into the Mechanism of Type VII Secretion System
Source: PLoS One. 2011 Nov 30;6(11):e27980. doi: 10.1371/journal.pone.0027980 (PMC3227618; doi:10.1371/journal.pone.0027980)
Supplement: Table S1 — List of mycobacterial species and the number of orthologs of the ESX-1 components. (PDF) [file pone.0027980.s005.pdf]

Table S1: List of mycobacterial species and the number of orthologs of the ESX-1 components*.

Organism name	Number of orthologs
***Mycobacterium tuberculosis* H37Rv**	**17**
Mycobacterium bovis AF2122 97	17
Mycobacterium marinum M	13
Mycobacterium leprae Br4923	12
Mycobacterium gilvum PYR GCK	12
Mycobacterium spyr1	12
Mycobacterium kms	11
Mycobacterium mcs	11
Mycobacterium smegmatis MC2 155	10
Mycobacterium jls	10
Mycobacterium vanbaalenii PYR1	9
Mycobacterium avium 104	0
Mycobacterium abscessus ATCC 9977	0
Mycobacterium ulcerans Agy99	0

* Experimentally identified to be associated to ESX-1 secretion pathway of *Mycobacterium tuberculosis* H37Rv.
