# Supplementary material for: Computational Analysis of the ESX-1 Region of Mycobacterium tuberculosis: Insights into the Mechanism of Type VII Secretion System
Source: PLoS One. 2011 Nov 30;6(11):e27980. doi: 10.1371/journal.pone.0027980 (PMC3227618; doi:10.1371/journal.pone.0027980)
Supplement: Table S2 — Summary of the predicted domains, transmembrane helices, 3D folds, presence of signal peptides and glycosylation sites in ESX-1 components. (PDF) [file pone.0027980.s006.pdf]

Table S2: Summary of the predicted domains, transmembrane helices, 3D folds, presence of signal peptides and glycosylation sites in ESX-1 components.

Component	Length (aa)	Domain	Probable position of tansmembrane helices	3D fold	Signal peptide	Probable position of glycosylation site(s)
Rv3866	283	DNA-binding	-	-	-	-
Rv3868	573	ATP-binding	-	TPR fold (N- terminal), fold similar to AAA domain (C-terminal)	-	-
Rv3869	480	-	40-60	-	-	144,148,152
Rv3870	747	FtsK	41-64 and 68-92	Fold similar to FtsK domain	-	-
Rv3871	591	FtsK	-			-
Rv3872	99	PE	-	Fold similar to PE family proteins	-	-
Rv3873	368	PPE	-	Fold similar to PPE family proteins	-	182, 183, 191, 194, 195, 196, 206, 208
Rv3874 (CFP-10)	100	ESAT-6 like	-	ESAT-6 like fold	-	-
Rv3875 (ESAT-6)	95	ESAT-6 like	-	ESAT-6 like fold	-	-
Rv3876	666	FlhG	-	Fold similar to ParA family proteins (C- terminal)	-	-
Rv3877	511		144-166, 171-190, 197-219, 229-246, 258-280, 284-306, 366-388, 392-414, 419-441, 445-467, and 480-502	Ubiquitin-like fold (N-terminal)	-	-
Rv3883c (MycP1)	446	Subtilisin	419-439	Fold similar tosubtilisin-like serine proteases	Yes	-
Rv3882c	462	-	9-29 and 33-53	-	Yes	-
Rv3615c (EspC)	103	-	-	ESAT-6 like fold	-	-
Rv3616c (EspA)	412	-	-	Collagen like triple helical fold (C- terminal)	-	-
Rv3881c (EspB)	460	-	-	Helix-turn-helix fold similar to PE family proteins followed by fold similar to PPE family proteins	-	-
Rv3879c	729	-	-	Fibrinogen-like fold	-	-
