# Supplementary material for: Computational Analysis of the ESX-1 Region of Mycobacterium tuberculosis: Insights into the Mechanism of Type VII Secretion System
Source: PLoS One. 2011 Nov 30;6(11):e27980. doi: 10.1371/journal.pone.0027980 (PMC3227618; doi:10.1371/journal.pone.0027980)
Supplement: Table S3 — Genes constituting the compositionally distinct islands that harbor (A) ESX-1 gene cluster and, (B) a part of the MCE Cluster 1 region. (PDF) [file pone.0027980.s007.pdf]

Table S3: Details of the genes constituting the compositionally distinct islands that harbor (A) ESX-1 gene cluster and, (B) a part of the MCE Cluster 1 region. The genes of the ESX-1 gene cluster and MCE Cluster 1 region being highlighted in blue and orange respectively.

(A)

PROTEIN ID	NAME	ANNOTATION
Rv3864	-	hypothetical protein
Rv3865	-	hypothetical protein
Rv3866	-	hypothetical protein
Rv3867	-	hypothetical protein
Rv3868	-	hypothetical protein
Rv3869	-	hypothetical protein
Rv3870	-	transmembrane protein
Rv3871	-	hypothetical protein
Rv3872	PE35	PE family-related protein
Rv3873	PPE68	PPE family protein
Rv3874	esxB	10 kDa culture filtrate antigen EsxB
Rv3875	esxA	6 kDa early secretory antigenic target ESXA (ESAT-6)
Rv3876	-	hypothetical protein
Rv3877	-	transmembrane protein
Rv3878	-	hypothetical protein
Rv3879c	-	hypothetical protein
Rv3880c	-	hypothetical protein
Rv3881c	EspB	hypothetical protein
Rv3882c	-	hypothetical protein
Rv3883c	mycP1	membrane-anchored mycosin MYCP1 (serine protease) (subtilisin-like protease) (subtilase-like) (mycosin-1)

PROTEIN ID	NAME	ANNOTATION
Rv3884c	-	CBXX/CFQX family protein
Rv3885c	-	hypothetical protein
Rv3886c	mycP2	alanine and proline rich membrane-anchored mycosin
Rv3887c	-	transmembrane protein
Rv3888c	-	hypothetical protein
Rv3889c	-	hypothetical protein
Rv3890c	esxC	ESAT-6 like protein ESXC (ESAT-6 like protein 11)
Rv3891c	esxD	ESAT-6 like protein EsxD
Rv3892c	PPE69	PPE family protein
Rv3893c	PE36	PE family protein
Rv3894c	-	hypothetical protein
Rv3895c	-	hypothetical protein
Rv3896c	-	hypothetical protein
Rv3897c	-	hypothetical protein
Rv3898c	-	hypothetical protein
Rv3899c	-	hypothetical protein
Rv3900c	-	hypothetical protein
Rv3901c	-	hypothetical protein
Rv3902c	-	hypothetical protein

(B)

PROTEIN ID	NAME	ANNOTATION
Rv0152c	PE2	PE family protein
Rv0153c	ptbB	phosphotyrosine protein phosphatase PTPB (protein-tyrosine-phosphatase) (PTPase)
Rv0154c	fadE2	acyl-CoA dehydrogenase FADE2
Rv0155	pntAa	NAD(P) transhydrogenase subunit alpha
Rv0156	pntAb	NAD(P) transhydrogenase subunit alpha
Rv0157	pntB	NAD(P) transhydrogenase subunit beta
Rv0158	-	TetR family transcriptional regulator
Rv0159c	PE3	PE family protein
Rv0160c	PE4	PE family protein
Rv0161	-	oxidoreductase
Rv0162c	adhE1	zinc-type alcohol dehydrogenase E subunit
Rv0163	-	hypothetical protein
Rv0164	TB18.5	hypothetical protein
Rv0165c	-	GntR family transcriptional regulator
Rv0166	fadD5	acyl-CoA synthetase
Rv0167	yrbE1A	integral membrane protein YRBE1A
Rv0168	yrbE1B	integral membrane protein YRBE1B
Rv0169	mce1A	MCE-family protein MCE1A
Rv0170	mce1B	MCE-family protein MCE1B
Rv0171	mce1C	MCE-family protein MCE1C

PROTEIN ID	NAME	ANNOTATION
Rv0172	mce1D	MCE-family protein MCE1D
Rv0173	lprK	MCE-family lipoprotein LprK
Rv0174	mce1F	MCE-family protein MCE1F
