# Supplementary material for: Computational Analysis of the ESX-1 Region of Mycobacterium tuberculosis: Insights into the Mechanism of Type VII Secretion System
Source: PLoS One. 2011 Nov 30;6(11):e27980. doi: 10.1371/journal.pone.0027980 (PMC3227618; doi:10.1371/journal.pone.0027980)
Supplement: Table S4 — List of gene components experimentally identified to be involved in ESX-1 secretion pathway. The genes and the corresponding protein names are taken from the TubercuList database (http://tuberculist.epf1.ch/). (PDF) [file pone.0027980.s008.pdf]

Table S4: List of gene components experimentally identified to be involved in ESX-1 secretion pathway. The genes and the corresponding protein names are taken from the TubercuList database (http://tuberculist.epf1.ch/).

**Gene name**	**Synonym code**	**Protein name**
espG1	Rv3866	EspG1
eccA1	Rv3868	EccA1
eccB1	Rv3869	EccB1
eccCa1	Rv3870	EccCA1
eccCb1	Rv3871	EccCB1
PE35	Rv3872	-
PPE68	Rv3873	-
esxB	Rv3874	CFP-10
esxA	Rv3875	ESAT-6
espI	Rv3876	EspI
eccD1	Rv3877	EccD1
espK	Rv3879c	EspK
mycP1	Rv3883c	MycP1
eccE1	Rv3882c	EccE1
espB	Rv3881c	EspB
espC	Rv3615c	EspC
espA	Rv3616c	EspA
